# Non-immune targeting of CXCR3 compromises mitochondrial function and suppresses tumor growth in glioblastoma

**DOI:** 10.1038/s41420-025-02449-1

**Published:** 2025-04-04

**Authors:** Travis Yui Hei Chan, Bo Chen, Wanjun Tang, Henry Hei Chan, Yogesh K. H. Wong, Ethan C. L. Wong, Junbo Liao, Anson Cho-Kiu Ng, Jenny Sum Yee Wong, Gilberto Ka-Kit Leung, Karrie M. Kiang

**Affiliations:** 1 Department of Surgery, School of Clinical Medicine, LKS Faculty of Medicine, The University of Hong Kong, Hong Kong, China; 2 The State Key Laboratory of Brain and Cognitive Sciences, The University of Hong Kong, Hong Kong, China

**Keywords:** Translational research, Cancer metabolism

## Abstract

The chemokine receptor CXCR3 is traditionally recognized for its role in immune cell trafficking. However, emerging evidence suggests that its functions may extend beyond the immune system, particularly in cancer, where its roles remain to be elucidated. In this study, we demonstrated that CXCR3 expression correlates with glioblastoma (GBM) grading, with CXCR3-A isoform being associated with poorer patient prognosis compared to CXCR3-B. Ablation of both CXCR3 isoforms significantly impaired GBM cell proliferation, migration, and tumor growth both in vitro and in immunodeficient mice. To elucidate the mechanistic role of CXCR3, we conducted transcriptomic profiling of tumor xenografts, revealing that CXCR3 depletion would disrupt mitochondrial homeostasis. This was further supported by our findings that CXCR3 would localize to the mitochondrial membrane, and that inhibition of CXCR3 would lead to mitochondrial depolarization and increased reactive oxygen species production. Notably, activation of phosphorylated-STAT3 rescued cell viability in CXCR3-depleted cells, suggesting that CXCR3 may modulate mitochondrial function through a STAT3-dependent mechanism, consistent with the known functional role of STAT3 in maintaining mitochondrial redox balance. Furthermore, treatment with the selective CXCR3 antagonist AMG487 reduced tumor growth and disrupted mitochondrial function in vitro, in vivo, and in patient-derived GBM stem cells. Our findings reveal CXCR3 as a previously unrecognized regulator of mitochondrial function in cancer cells, positioning the CXCR3-mitochondrial signaling axis as a promising therapeutic target for GBM.

**Figure:**
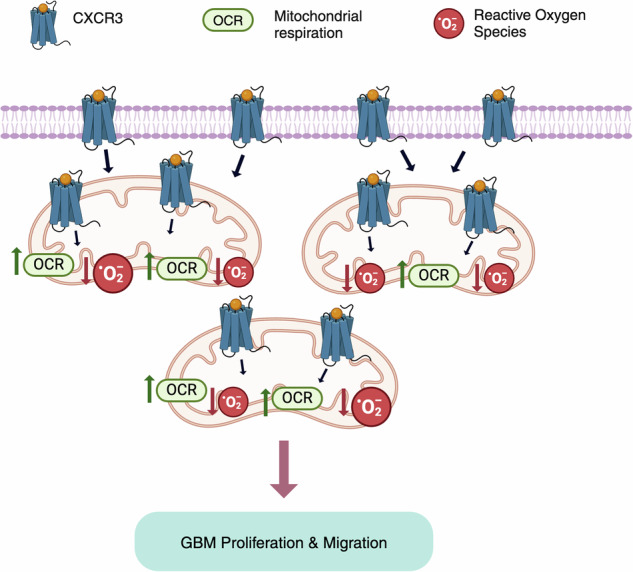
Chemokine receptors are well-established mediators of inflammatory responses, emerging evidence suggests that these receptors may play roles beyond the immune system. In this study, we have demonstrated that CXCR3 would localize to the mitochondrial membrane and exert a previously unrecognized function in regulating cancer metabolism and mitochondrial function. Figure created using BioRender (https://biorender.com).

Chemokine receptors are well-established mediators of inflammatory responses, emerging evidence suggests that these receptors may play roles beyond the immune system. In this study, we have demonstrated that CXCR3 would localize to the mitochondrial membrane and exert a previously unrecognized function in regulating cancer metabolism and mitochondrial function. Figure created using BioRender (https://biorender.com).

## Introduction

CXCR3 is a G protein-coupled receptor expressed on the cell surface of various cell types, including both immune cells and non-immune populations [1]. In immune cells such as T cells and dendritic cells, CXCR3 functions as a chemokine receptor, orchestrating the recruitment of these cells to sites of inflammation by recognizing its ligands CXCL9, CXCL10, and CXCL11 [2]. Current literature indicates that CXCR3 plays an intricate role in cancer cells, with evidence suggesting it can both promote and inhibit tumor progression depending on the balance between its isoforms CXCR3-A and CXCR3-B [3]. The CXCR3-A isoform is associated with pro-tumor effects, such as enhanced angiogenesis and tumor cell proliferation, whereas CXCR3-B confers anti-tumor properties through inhibiting angiogenesis and inducing apoptosis in malignant cells [4–6]. This dichotomous role of CXCR3 isoforms has been demonstrated across several cancer types, including breast and lung cancers, where the balance between isoforms appears critical for determining net tumor growth [7].

A major gap exists in our understanding of CXCR3’s non-immune functions in cancer. While its role in immune cell trafficking through chemokine activities is well characterized, research into its downstream non-immune mechanisms has largely been confined to mainstream pathways such as PI3K/AKT or SRC-RAS-ERK [5, 8]. However, these important studies have yet to offer translational impact; instead, emerging evidence has identified an alternative, mitochondrial dysfunction pathway, where CXCR3 ablation may protect against steatohepatitis by impairing mitochondrial ATP respiration and disrupting the balance of fission and fusion protein expression [9]. Elucidating the potential role of this mechanistic pathway in glioblastoma (GBM) pathogenesis could reveal novel therapeutic targets, given the profound impact that mitochondrial functions may have in this highly malignant neoplasm.

The present study aimed to interrogate CXCR3’s potential non-immune functions in GBM. We report for the first time that CXCR3 localizes to the mitochondria of GBM cells and plays a critical role in regulating mitochondrial function and tumor growth. Most importantly, targeting CXCR3 significantly suppresses GBM growth through non-immune mechanisms. Our findings underscore the critical role of CXCR3 in GBM progression and highlight the therapeutic potential of targeting this receptor to mitigate tumor growth and improve patient outcomes.

## Results

### CXCR3 is significantly overexpressed in gliomas and correlates with poor prognosis

**Fig. 1:**
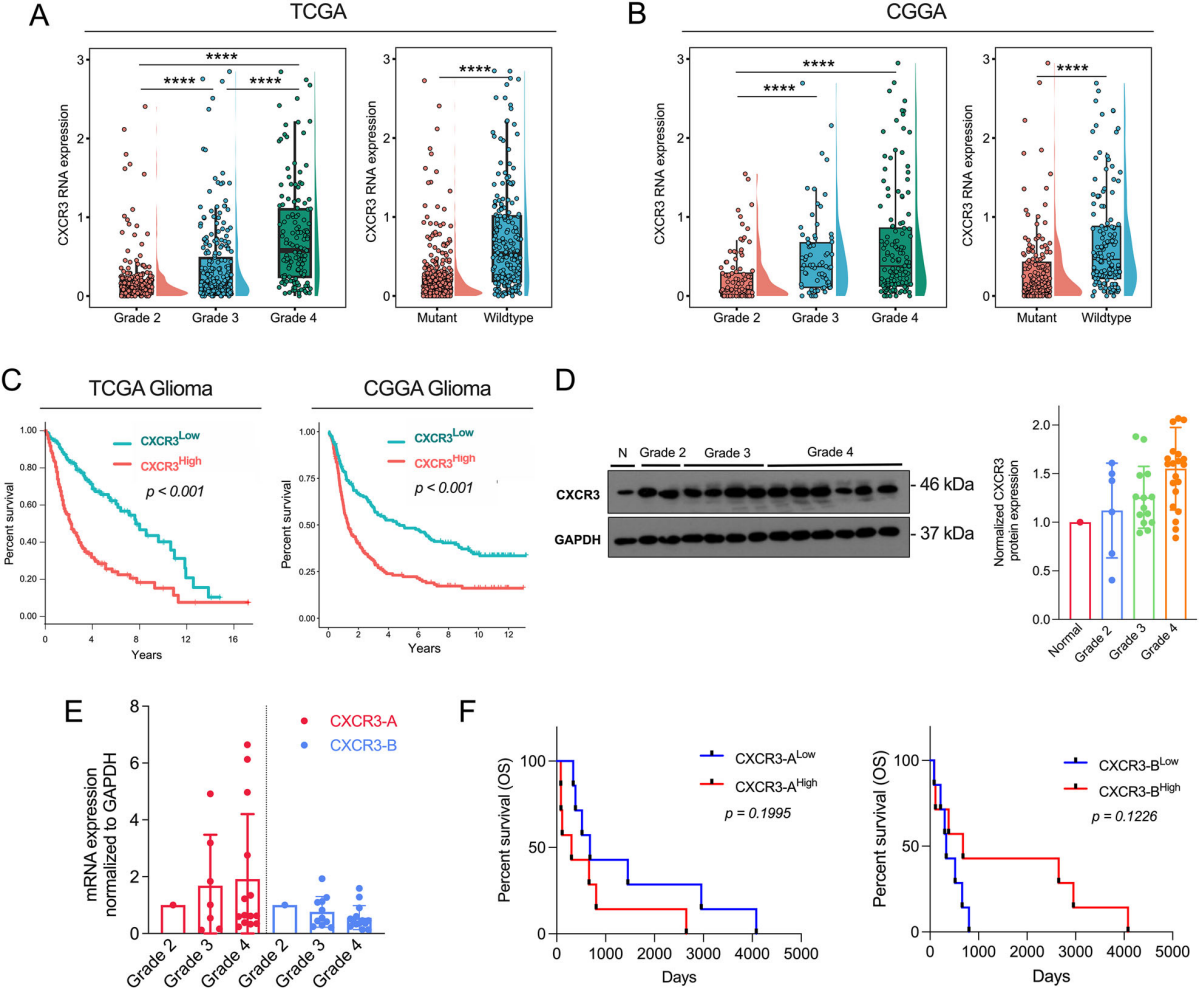
CXCR3 is overexpressed in gliomas and associated with poorer survival. **A**, **B** The mRNA expression of CXCR3 in gliomas from two publicly available databases stratified by WHO grade and IDH mutation status. (CGGA, *n* = 320; TCGA, *n* = 650). **C** Kaplan–Meir survival analysis after stratifying patients into CXCR3 high and CXCR3 low groups based on median mRNA expression from TCGA and CGGA databases. (CGGA, CXCR3 high, *n* = 160; CXCR3 low, *n* = 169) (TCGA, CXCR3 high, *n* = 321; CXCR3 low, *n* = 324). **D** Western blot analyses and quantification of CXCR3 protein level in Grade 2–4 QMH glioma patients. **E** qPCR analyses of CXCR3-A and CXCR3-B mRNA expression levels in Grade 2–4 QMH glioma patients. **F** Kaplan–Meir survival analysis of QMH GBM patients into CXCR3-A and CXCR3-B high and low groups based on median mRNA expression level of GBM clinical samples (*n* = 7 per group). Error bars indicate mean ± SD. *p < 0.05, **p < 0.01, ***p < 0.001,****p < 0.0001, ns (no statistical significance).

Transcriptomic analysis of gliomas from the TCGA and Chinese Glioma Genome Database (CGGA) databases revealed progressive upregulation of CXCR3 mRNA expression across WHO grades 2–4, inclusive of both IDH-wildtype and IDH-mutant gliomas. CXCR3 expression was significantly associated with IDH-wildtype gliomas, which is a prognostic marker for poor clinical outcome (Fig. 1A, B). To gain insights into the clinical significance of CXCR3, data from both databases confirmed that high CXCR3-expressing gliomas had significantly lower overall survival rate (Fig. 1C). We also confirmed overexpression of CXCR3 in an expanded cohort of 45 glioma patients, where protein expression increased by 41.6% in grade 4 GBM compared to normal brain tissue, and exhibited a graduate increase from low to high-grade gliomas (Fig. 1D). Consistent with these findings, qPCR analysis on grade 2–4 gliomas from our in-house tumor samples revealed upregulation of the oncogenic CXCR3-A isoform (1.917-fold change), while demonstrating downregulation of the anti-oncogenic CXCR3-B isoform (0.558-fold change) (Fig. 1E). Kaplan–Meier survival analysis using GBM samples (grade 4, IDH-wildtype) from our 14 in-house specimens demonstrated significant correlation of CXCR3-A and CXCR3-B with poorer (log-rank *p* = 0.1995) and better survival (log-rank *p* = 0.1226) outcome, respectively (Fig. 1F).

### Knockdown of CXCR3 impedes tumor growth

**Fig. 2:**
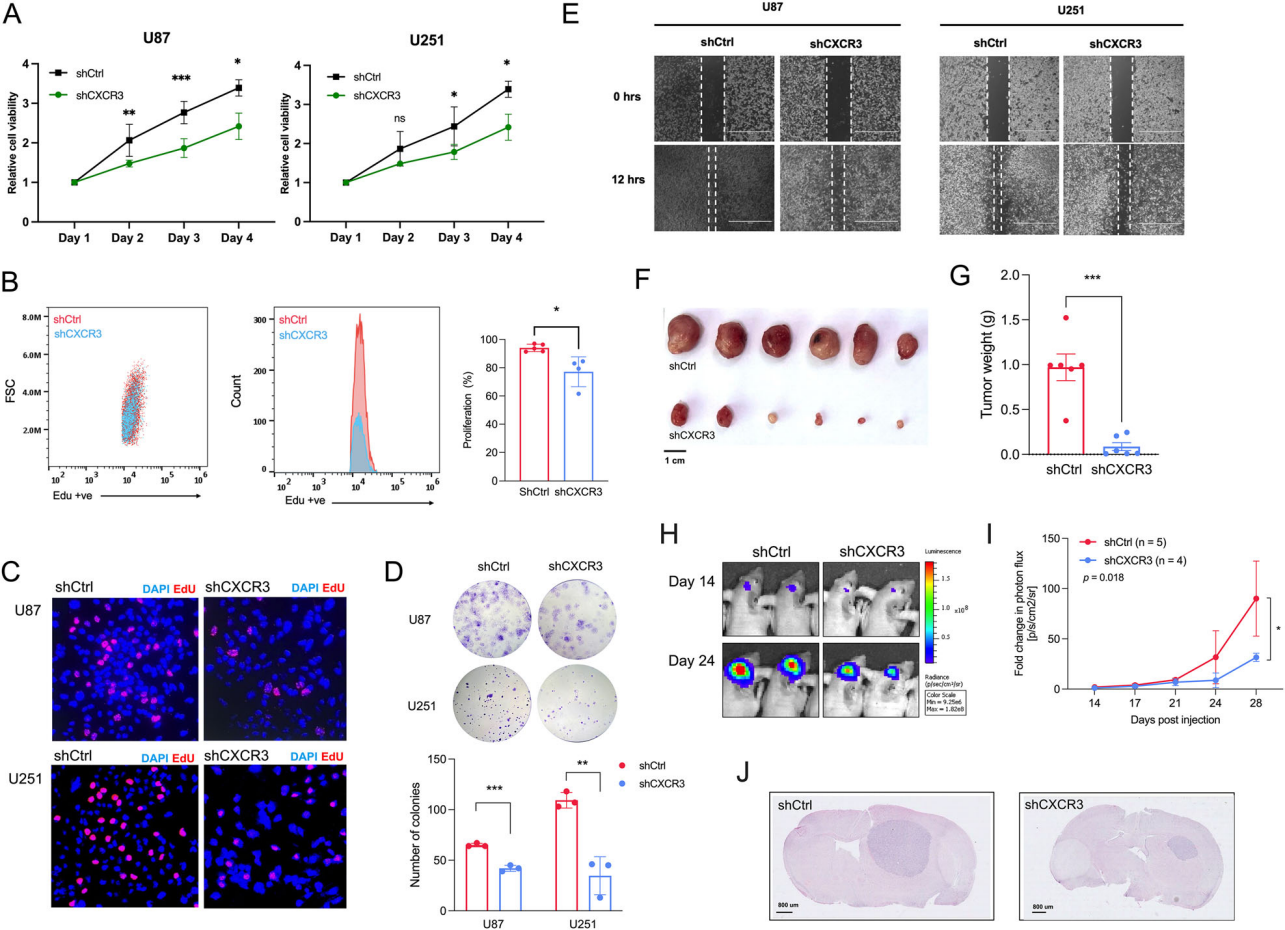
Knockdown of CXCR3 reduces tumor cell proliferation. **A** MTT assays of U87 and U251 cells with CXCR3 knockdown (shCXCR3) from day 1 to day 4 compared to control (shCtrl). **B** EdU incorporation assay showed decreased U87 EdU^+^ proliferating cells following CXCR3 knockdown measured by flow cytometry (left). Quantification of signal intensity (right). **C**. Representative fluorescence images of EdU stained U87 and U251 cells. EdU (red); DAPI (blue). Original magnification ×40 **D**. Representative images (top) and quantification of colonies (bottom) formed after 14 days of incubation. **E** Representative images of wound simulation assay after 12 hours of incubation. Original magnification: ×4; Scale bar: 100 mm. **F**, **G** Representative images and tumor weight of U87 subcutaneous xenografts from nude mice at day 28 after tumor injection (*n* = 6). **H**, **I** Representative image and quantification of bioluminescence intensity in U87 orthotopic xenograft expressing shCXCR3 (*n* = 5) compared to shCtrl control (*n* = 4). The bioluminescence signal was measured twice per week at indicated intervals. Photon flux (p/s/cm^2^/sr) was normalized at day 1 and presented as fold change at day 14, 17, 21, 24, and 28. **J**. Representative H&E staining of orthotopic implanted U87 cells after mice sacrifice at day 28 at experimental endpoint, Scale bar 800 μm. Error bars indicate mean ± SD. *p < 0.05, **p < 0.01, ***p < 0.001, ****p < 0.0001, ns (no statistical significance). **G**, **I** Error bars indicate mean ± SEM.

To investigate the functional role of tumor CXCR3 in GBM pathogenesis, we generated a stable knockdown of CXCR3 in U87 cells and confirmed the depletion of CXCR3 protein expression. MTT proliferation assay, which measures metabolic activity as a readout of cellular viability, revealed that knockdown of CXCR3 reduced cell viability compared to control after 72 hours (Fig. 2A). Consistently, cell proliferation was impeded following CXCR3 depletion with a significant reduction of EdU incorporation (Fig. 2B, C). CXCR3 knockdown also had inhibitory effects on both clonogenic growth, which is indicative of tumorigenic potential, and migratory ability of tumor cells (Fig. 2D, E). To exclude the immunological effects of CXCR3 mediated by T cells, we used athymic nude mice with T cell deficiency as a model to investigate the functional role of CXCR3 in tumor cells. 28 days after subcutaneous injection of tumor cells with shCtrl and shCXCR3, tumor growth in CXCR3 knockdown group was profoundly suppressed (*p* = 0.002) (Fig. 2F, G). To further confirm our observation in a more disease-relevant tumor microenvironment, we orthotopically implanted CXCR3 control and knocked down U87 cells into the intracranial region. Consistently, tumor growth was significantly reduced after CXCR3 knockdown at 24 days post injection (*p* = 0.0180) (Fig. 2H–J). Our data suggest that tumor CXCR3 per se may play a role in promoting tumor growth in an immunodeficient setting.

### CXCR3 knockdown alters the metabolic program and impairs mitochondrial function

**Fig. 3:**
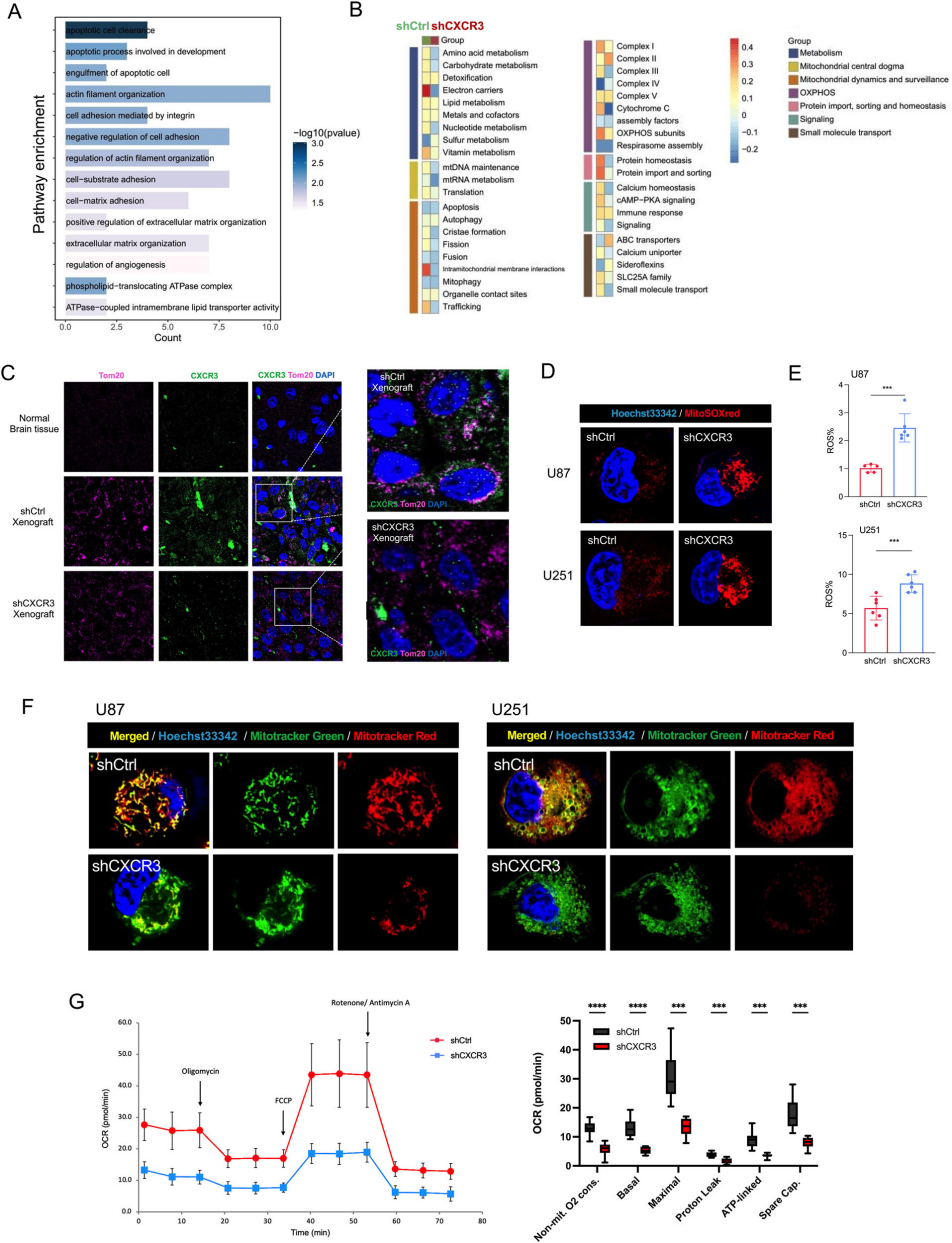
CXCR3 knockdown contributes to mitochondrial dysfunction. **A** Pathway enrichment analysis using RNA sequencing data from orthotopic xenografts illustrating key signaling pathways involved after CXCR3 ablation. *n* = 3 per group. **B** Heatmap analysis highlighting changes in expression of mitochondrial processes in shCtrl and shCXCR3 xenografts. **C** Representative fluorescence images from orthotopic xenografts showing the subcellular localization of CXCR3 receptor expression in normal brain tissue. Original magnification: ×40. **D** Representative fluorescence images stained with mitoSOX (red) and Hoechst 33342 (blue). **E** Flow cytometry analysis of mitochondrial reactive oxygen species production from U87 and U251 cells expressing shCXCR3 compared to control. Red indicates the MitoSOX red stain, which detects superoxide production within the mitochondria of live cells. **F** Representative fluorescence images in U87 and U251 cells. Green indicates the MitoTracker green dye, which labels mitochondria and provides a measure of total mitochondrial mass within live cells. Red indicates the Mitotracker red CMXROS dye, which accumulates in mitochondria based on their membrane potential. Blue indicates hoechst 33342. Reduced Mitotracker red staining indicates dysfunctional mitochondria with mitochondrial depolarization. **G** Seahorse mito stress analysis of mitochondrial respiration in U87 cells. (Top) Oxygen Consumption rate (OCR) of U87 cells expressing shCtrl and shCXCR3 under basal conditions, followed by sequential addition of mitochondrial inhibitors (right). Quantification of key respiratory parameters: Non-mit O2 cons non-mitochondrial O2 consumption, Basal basal respiration, Maximal maximal respiration, ATP-linked ATP-linked respiration, Spare Cap. spare capacity. *n* = 8 per group. Error bars indicate mean ± SD. *p < 0.05, **p < 0.01, ***p < 0.001, ****p < 0.0001, ns (no statistical significance).

Next, we investigated the possible impacts of CXCR3 depletion on downstream pathway. Transcriptomic profiling was performed on shCtrl and shCXCR3 orthotopic xenografts. Pathway enrichment of differentially expressed genes revealed upregulation of apoptotic clearance upon CXCR3 ablation. Since mitochondria are central to the intrinsic pathway of apoptosis [10], we surmised that the activation of apoptotic cell clearance could be mediated through mitochondrial cell death (Fig. 3A). To assess mitochondrial function and to determine the associated metabolic changes, mitochondrial-associated genes were selected for further analysis. We identified downregulation of genes associated with mitochondrial function and cellular metabolism in CXCR3-depleted cells (Fig. 3B). In contrast to previous reports indicating the exclusive expression of CXCR3 on the cellular membrane [6], we discovered for the first time that CXCR3 expression was colocalized with mitochondrial protein Tom20 in GBM cells, suggesting the possible modulation of mitochondrial function by CXCR3 in promoting tumor growth (Fig. 3C).

In line with this, CXCR3 knockout increased the production of mitochondrial superoxide (Fig. 3D, E) as well as depolarization of mitochondrial membrane potential (Fig. 3F), indicating mitochondrial function impairment when CXCR3 is ablated. Mitochondrial function was further evaluated by cell mito stress assay. Mitochondrial respiration profile was generated upon sequential injection of respiratory modulators targeting different parts of the electron transport chain. We noticed significant reductions in maximal respiration as a result of CXCR3 knockdown, indicating the critical role of CXCR3 in maintaining mitochondrial function (Fig. 3G). Taken together, our multi-parametric evaluation of CXCR3-modulated mitochondrial physiology has provided novel mechanistic insights into how CXCR3 may support the heightened metabolic and biosynthetic needs of GBM through the coordinated control of mitochondrial homeostasis.

### CXCR3 regulates mitochondrial function through p-STAT3

**Fig. 4:**
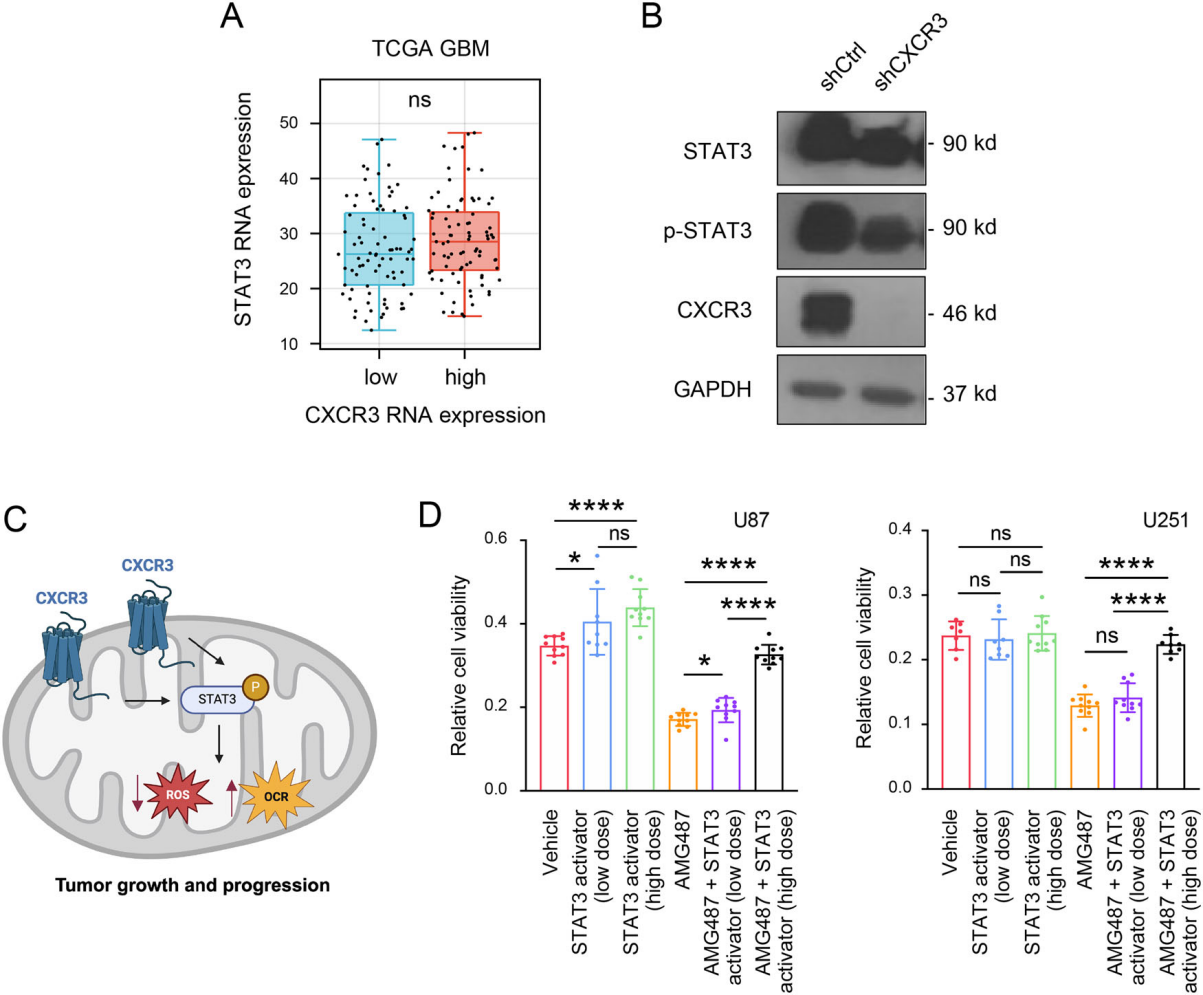
CXCR3 regulates mitochondrial function through p-STAT3 signaling. **A** The mRNA expression of STAT3 in CXCR3^high^ and CXCR3^low^ GBMs from a publicly available database. (TCGA, *n* = 166). **B** Western blot analysis of total STAT3 and Phospho-STAT3 (p-STAT3) expression in U87 cells expressing shCtrl and shCXCR3. **C** Relative cell viability in U87 and U251 cells treated with the CXCR3 inhibitor AMG487 for 72 hours, withglioblastoma stem cell or without the addition of low-dose or high-dose STAT3 activator. The STAT3 activator was used to rescue the effects of CXCR3 inhibition on downstream STAT3 activity. **D** A hypothetical diagram depicting the proposed mechanism whereby increased CXCR3 activation may lead to enhanced phosphorylation and activation of STAT3 in the mitochondria, which in turn promotes STAT3-mediated redox balance and enhances cancer metabolism as indicated by the increased OCR in GBM. Figure created using BioRender (https://biorender.com). (ROS reactive oxygen species, OCR oxygen consumption rate) Error bars indicate mean ± SD. *p < 0.05, **p < 0.01, ***p < 0.001, ****p < 0.0001, ns (no statistical significance).

Our analysis of gene expression patterns showed dysregulations of apoptotic and cytoskeletal pathways when CXCR3 was silenced, prompting further investigation into the downstream processes. STAT3, a key transcription factor known for its role in mitochondrial functioning and cell death in cancer [11], has been previously reported to be involved in the CXCR3 regulation by which CXCR3 ligands induce STAT3 activation [11]. While transcriptomics analysis revealed that STAT3 mRNA expression was unaffected by CXCR3 expressions (Fig. 4A, B), we found that knockdown of CXCR3 instead reduced activation of STAT3 (i.e., p-STAT3 expression) (Fig. 4B). To determine if p-STAT3 mediates CXCR3’s effects on GBM proliferation, we performed a rescue experiment. Treating CXCR3-knockout cells with a STAT3 phosphorylation activator led to a dose-dependent viability increase by MTT assay (Fig. 4C). These results implicate p-STAT3 as a functional CXCR3 signaling target in GBM. Given p-STAT3’s established roles in modulating mitochondria, apoptosis, and cytoskeleton functioning, it is likely that its dysregulation may underlie metabolic and phenotypic changes associated with the CXCR3 expression profile. Together, the data suggest that CXCR3 may regulate mitochondrial physiology and GBM pathogenesis, at least partly, through p-STAT3-dependent mechanisms (Fig. 4D).

### CXCR3 inhibition by AMG487 in GBM reduces tumor growth

**Fig. 5:**
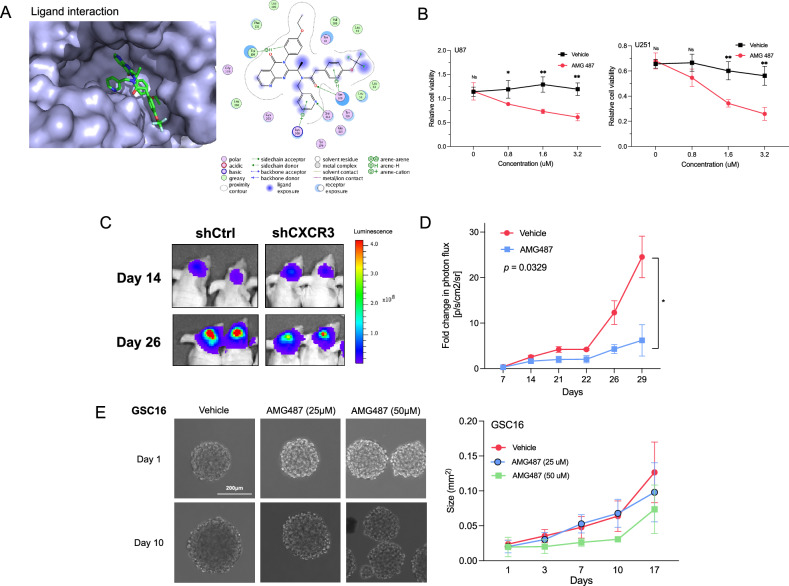
Pharmacological inhibition of CXCR3 by AMG487 suppresses tumor growth. **A** Structural modeling analysis depicting the binding interactions between the small molecule CXCR3 inhibitor AMG487 and the CXCR3 receptor protein. **B** Relative cell viability of U87 and U251 cells following 72-hour treatment with increasing concentrations of AMG487 (0, 0.8, 1.6, 3.2 μM) compared to control. Error bars indicate mean ± SD. *p < 0.05, **p < 0.01^,^ **C**, **D** Representative image and quantification of bioluminescence intensity in orthotopic xenograft in nude mice bearing U87 cells expressing shCXCR3 (*n* = 5) compared to control (*n* = 4) after AMG487 treatment (10 mg/kg). Bioluminescence intensity was measured twice per week at indicated intervals. Photon flux (p/s/cm^2^/sr) was normalized on day 1 and presented as fold change on days 7, 14, 21, 22, 26, and 29. Error bars indicate mean ± SEM. **E** Representative brightfield images and quantification of glioblastoma stem cell (GSC) spheroid size (mm^2^) after 17 days of vehicle (DMSO), low (25 μM) and high (50 μm) dose AMG487 treatment, scale bar 200 μm.

The clinical relevance of CXCR3 in GBM progression was explored utilizing the selective CXCR3 inhibitor, AMG487, which has a high affinity to the CXCR3 protein (Fig. 5A). In vitro, after administration with AMG487 for 72 hours, MTT analysis showed that there was a dose-dependent decrease in cell viability (Fig. 5B). These results were further validated with an in vivo orthotopic mouse model, where AMG487 treatment led to a significant decrease in tumor intensity and size when compared to controls (Fig. 5C, D). Finally, AMG487 was given to primary glioblastoma stem cell culture (GSC16), which also showed a dose-dependent reduction in cell size relative to controls (Fig. 5E).

### CXCR3 inhibition by AMG487 can reduce mitochondrial dysfunction

**Fig. 6:**
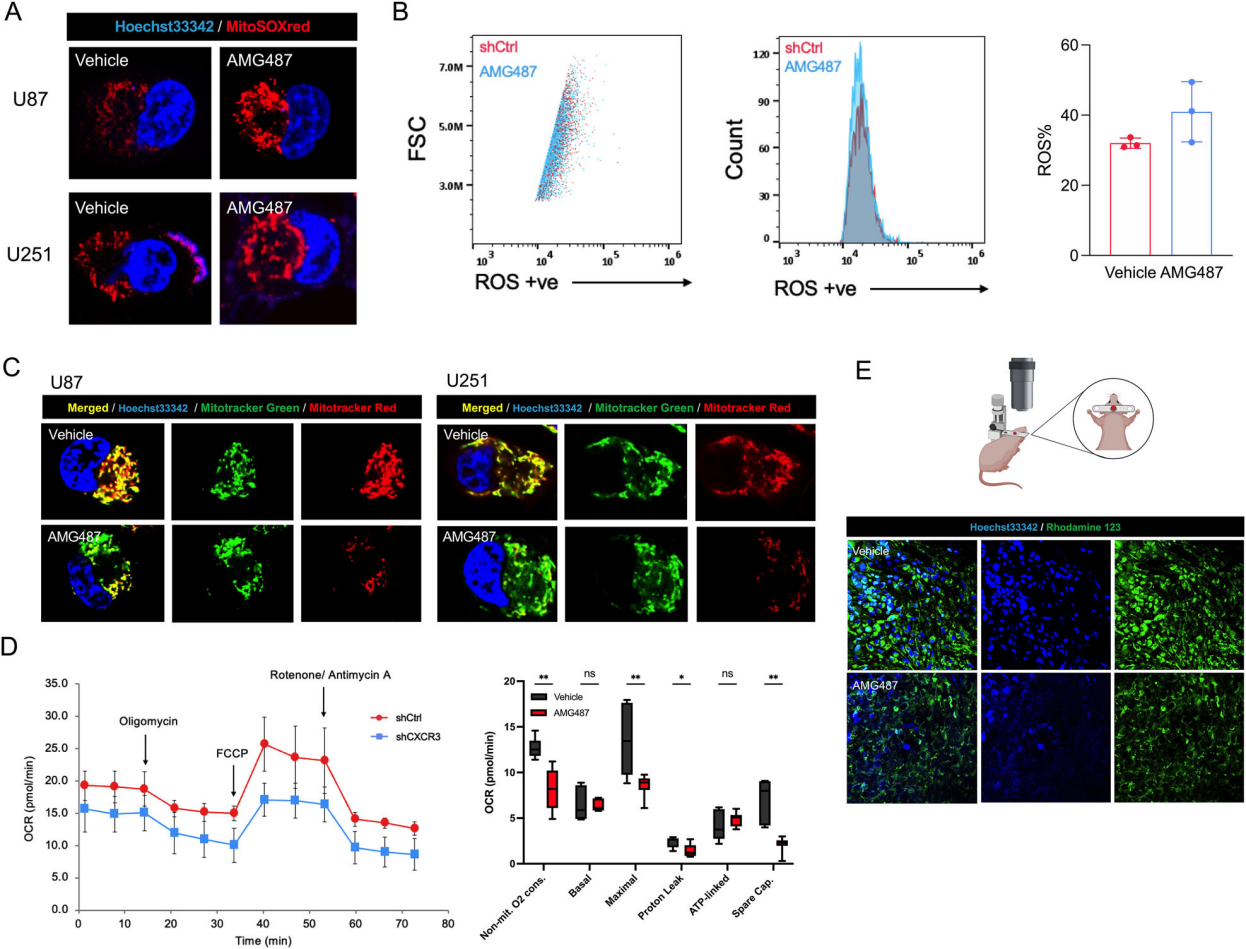
Pharmacological inhibition of CXCR3 by AMG487 compromises mitochondrial function. **A**, **B** Representative fluorescence images and flow cytometry analysis of mitochondrial ROS production, MitoSOX red (red) and hoechst 33342 (blue) from U87 and U251 cells treated with AMG487 (dose). **C** Representative fluorescence images from confocal imaging in cells treated with vehicle (DMSO) and AMG487 (dose) stained with Mitotracker green, Mitotracker red, and hoechst 33342 (blue). **D** Seahorse analysis of mitochondrial respiration in U87 cells. (left) and OCR of U87 cells treated with vehicle (DMSO) and AMG487 under basal conditions, followed by sequential addition of mitochondrial inhibitors (right). *n* = 7 per group. **E** Schematic diagram illustrating the two-photon imaging approach used in the study. Representative two-photon microscopy images of the orthotopic U87 xenograft in mice treated with AMG487 and vehicle (DMSO) for 21 days. Green indicates the rhodamine 123 stain, which labels mitochondrial membrane potential within the tumor cells, hoechst 33342 (blue).

Confocal microscopy and flow cytometry analyses revealed that ROS production significantly increased following AMG487 inhibition (Fig. 6A, B). There was a notable decrease in membrane potential observed after treatment with AMG487 (Fig. 6C). Furthermore, mitochondrial function as indicated by OCR (oxygen consumption rate) demonstrated a significant decrease in mitochondrial respiration upon AMG487 treatment, particularly for maximal respiration (Fig. 6D). In vivo two-photon imaging of mice with orthotopic xenograft treated with AMG487 showed a reduction in mitochondrial membrane potential compared to the control group (Fig. 6E). These findings collectively indicate that CXCR3 inhibition by AMG487 is potential promising to suppress tumor growth by impairing mitochondrial function.

## Discussion

Recent research on CXCR3 has predominantly focused on its role within the immune system, leaving its non-immune functions relatively underexplored [5]. Our study provides novel insights into the roles of CXCR3 beyond immune cell trafficking. While a previous histological study established that GBM overexpresses CXCR3 compared to lower-grade diffuse astrocytoma, we further validate that CXCR3 is positively correlated with glioma grade [12]. These findings are supported by data mining from two public databases. Notably, our study is the first to simultaneously demonstrate, using patient samples, that the CXCR3-A isoform is associated with poorer prognosis, whereas CXCR3-B correlates with better survival outcomes. This underscores the notions that it is the balance of CXCR3 isoform expression, rather than the overall CXCR3 levels, which significantly affects glioma progression, and that an imbalance in isoform expression may result in tumors with distinct prognostic and therapeutic responses. Consequently, targeted therapeutics should focus on shifting the balance of CXCR3 isoforms or selectively inhibiting or activating a specific isoform, as appropriate. This approach is similar to the therapeutic strategies employed for BCL-2 protein isoforms in various cancers, such as leukemia or lymphoma, where BH3-mimetics targets specific sites on different BCL-2 isoforms, depending on the cancer type [13]. In terms of CXCR3’s functional role in glioma, our findings build on previous research [14] by demonstrating that CXCR3 not only promotes glioma growth but also enhances cellular migration and colony formation. The observed tumor growth appears to be driven by increased GBM cell proliferation, consistent with findings in other cancers [15]. Overall, our data support the emerging view of CXCR3 as a critical tumor-expressed marker involved in multiple aspects of tumor pathogenesis [16].

A significant finding of our study is the elucidation of CXCR3’s impact on mitochondrial function in GBM cells. While mitochondrial dysfunction is increasingly recognized as a contributor to oncogenesis, the roles of mitochondria in cancer remain underexplored compared to their well-studied roles in inflammatory liver and skeletal muscle diseases [6, 17]. We demonstrated that CXCR3 knockdown reduces mitochondrial ROS production, which typically lowers oxidative stress levels associated with DNA damage, mutation rates, and oncogenic phenotypic changes in GBM [18]. Additionally, CXCR3 knockdown resulted in decreased mitochondrial membrane potential and respiration rates, both of which are key markers linked to apoptotic protein release and tumor cell ATP production [19, 20]. These findings suggest that CXCR3-mediated mitochondrial dysfunction is a critical driver of gliomagenesis by disrupting genomic integrity and bioenergetic balance. Moreover, we propose a functional pathway linking CXCR3 to GBM progression through phospho-STAT3-mediated mitochondrial signaling, as shown by the STAT3 activator Colivelin which rescued the effects of CXCR3 inhibition on cell viability. This pathway affects mitochondrial function by modulating electron transport chain pathways, as phospho-STAT3 is known to regulate mitochondrial signaling in cancer. While the roles of CXCR3, STAT3 phosphorylation, and mitochondrial modulation have been studied individually in various cancers, our study presents an integrated model that combines these elements to elucidate how aberrant CXCR3 signaling drives gliomagenesis [21].

AMG487 is an orally administered selective CXCR3 receptor antagonist, which inhibits the binding of CXCL10 and CXCL11 ligands to the CXCR3 receptor. A phase 1 clinical trial has demonstrated the efficacy of AMG487 in inflammatory diseases, including psoriatic arthritis [22]. In cancer research, AMG487 has shown efficacy in multiple preclinical studies involving metastatic osteosarcoma and metastatic breast cancer [23, 24]. Meanwhile, in glioma, AMG487 has been reported to prolong overall survival in tumor-bearing mice compared to controls [14]. Consistently, our findings demonstrate that AMG487 reduces GBM growth in both subcutaneous and intracranial mouse models, as well as in patient-derived stem cell cultures, highlighting its potential as a therapeutic agent. Interestingly, AMG487 exhibited a greater impact on mice with subcutaneous xenografts compared to those with orthotopic xenografts, possibly due to differences in drug delivery efficiency and tumor microenvironment characteristics, such as vascularization and blood-brain barrier permeability. The efficacy of AMG487 in GSCs is particularly significant, as GSCs are known for their resistance to conventional therapies and their role in tumor recurrence [25].

Our data also suggest that CXCR3 inhibition may disrupt pro-tumoral mitochondrial functions by modulating upstream pathways that regulate mitochondrial biology, rather than directly targeting mitochondrial pathways. From a translational perspective, direct targeting of mitochondria pathways has been found to be challenging due to potential severe systemic toxicities from broad metabolic perturbations and difficulties in dosage predictions [26]. In contrast, specifically targeting CXCR3 upstream of mitochondria regulation could achieve anti-tumor effects while minimizing toxicity risks by preserving normal mitochondrial homeostasis.

One limitation of the current study is the inability to interrogate the individual functions of the CXCR3-A and CXCR3-B isoforms due to the lack of commercially available isoform-specific antibodies for western blot analysis. As a result, we could not delineate the distinct functional roles of these isoforms. Similarly, re-expression of the CXCR3 knockdown cells to further verify our findings was not possible, due to the challenges associated with delivering the necessary constructs effectively into GBM cells. Future research should employ isoform-specific knockdown or overexpression approaches to provide more detailed mechanistic insight into the unique functions of each CXCR3 isoform. Another limitation is the use of immunodeficient mice which precluded exploration of any concomitant immunological role of CXCR3 in treatment response. These models lack the complexity of immune-tumor interactions present in immunocompetent hosts, potentially overlooking CXCR3’s role in modulating immune cell infiltration, activation, and function within the tumor microenvironment. Although a previous study indicated that CXCR3 antagonism does not promote immune cell migration to the glioma tumor site [14], future research investigating the effects of CXCR3 inhibition in combination with immunotherapies could provide valuable insights into potential synergistic therapeutic effects.

## Conclusions

This proof-of-concept study establishes a novel approach towards CXCR3 targeting in cancer treatment, encouraging further research on delineating the unique functions of CXCR3 isoforms through isoform-specific modulation techniques, such as RNAi or CRISPR, to selectively knockdown or express CXCR3-A and CXCR3-B. Optimizing CXCR3 antagonism in combination with standard therapies or immunotherapies in orthotopic glioma models will help clarify CXCR3’s role in modulating the tumor microenvironment and improve predictions of treatment responses. Lastly, emphasis should be placed on elucidating the precise molecular mechanisms by which CXCR3 modulates mitochondrial function through downstream effectors like STAT3, potentially revealing novel combinatory therapeutic targets.

## Materials and methods

### Cell lines and culture conditions

Human malignant glioma cell lines U87 and U251 were obtained from the American Type Culture Collection. Cells were routinely cultured in Minimum Essential Medium (Gibco), supplemented with 10% heat-inactivated fetal bovine serum (Gibco). Patient-derived GSCs were cultured in a serum-free medium enriched with a combination of growth factors, including hEGF (20 ng/mL, GIBCO), B27 (20 ng/mL), 1% N2, bFGF (20 ng/mL), and L-glutamine (2 mM). GSCs were cultured as spheroids using the AggreWell^TM^ system (StemCell Technologies). All the cells were kept in a humidified incubator at 37°C with 5% CO_2_. Selective CXCR3 inhibitor AMG487 (HY-15319) was procured from MedChemExpress and reconstituted with 10% DMSO.

### Establishment of stable knockdown cell lines

Stable knockdown of CXCR3 was achieved by employing lentiviral transduction of luciferase-expressing shRNA. The recombinant lentiviral vector (pGLV6/Firefly/Puro) was purchased from GenePharma and was transfected into HEK293T cells using the Lenti-vpak lentiviral packaging kit (Takara). U87 and U251 cells were transduced with lentivirus-containing media with 5 μg/mL polybrene added for 72 hours, followed by puromycin selection. The efficacy of gene silencing was assessed through qPCR and western blot analyses. The targeted sequences of control and CXCR3 in lentiviral vectors: shCtrl: 5’-GTTCTCCGAACGTGTCACGT-3’ and shCXCR3: 5’-GCCCTCTTCAACATCAACTTC-3’. The shRNA sequence recognizes a conserved region shared by both isoforms, resulting in concurrent silencing.

### Cell proliferation and clonongenic assay

Cell proliferation and viability were assessed by MTT (Thiazolyl blue tetrazolium bromide) (M5655, Sigma) and EdU incorporation assay kit (Ribobio). MTT or EdU reagents were added to each well at the experimental endpoint and incubated at 37 °C for 2 hours. For MTT assay, absorbance was measured using Varioskan LUX multimode microplate reader at 595 nm to determine the rate of cell proliferation. For EdU incorporation assay, cells were fixed with 4% paraformaldehyde in PBS. Subsequently, staining reagents were added according to manufacturer’s protocol, fluorescence staining was measured with confocal microscopy (Zeiss LSM 880) or flow cytometry (NovoCyte Quanteon/Advanteon). For the clonogenic assay, 500 cells/well were seeded in triplicates onto 6-well plates and maintained in culture for two weeks. Subsequently, cells were fixed with 75% alcohol and stained with 0.5% w/v solution of crystal violet. Images were acquired under brightfield illumination using Carl Zeiss AxioZoom.V16 light microscope.

### Wound healing cell migration assay

A total of 7 × 10^5^ cells were seeded onto a culture insert of the two-well µ-Dish (ibidi). The migration of cells towards the cell-free gap area was monitored at various time intervals over a 24-hour period. The distances were evaluated using Image J software.

### Immunoblotting

Protein lysates were extracted with RIPA lysis buffer with a protease inhibitor cocktail. The lysates were loaded onto SDS-PAGE gels and subjected to electrophoresis. The separated proteins were then transferred to PVDF membranes and blocked with a 5% non-fat milk solution in 5% TBST for 1 hour. For immunodetection, primary antibodies of CXCR3 (A11294, Abclonal), STAT3 (CST12640S), phospho-STAT3 (CST-9145S), and GAPDH (CST2118) were used. Detailed procedures of immunoblotting were performed as previously described [27].

### RT-qPCR

Total RNA was extracted using the RNAiso Plus kit (Takara). RNA was subsequently reverse-transcribed into cDNA using PrimeScript RT reagent kit with gDNA eraser (Takara). Quantitative PCR analysis was conducted using SYBR Green-based PCR kit (Takara) to measure mRNA expressions. See Supplementary Table 1 for primer sequences.

### Detection of mitochondrial membrane potential

In vitro mitochondrial membrane potential was measured with MitoTracker Green (20 nM, M7514) and MitoTracker Red (100 nM, M22425) (ThermoFisher) for 30 minutes at 37 °C. Cells were then washed twice with PBS and stained with Hoechst 33342 (5 µg/ul, ThermoFisher) for 15 minutes. Live images of the cells were captured with a confocal microscope (Zeiss LSM 880) and analyzed using Zeiss Zen Blue software. Cranial window was constructed in mice two weeks after orthotopic tumor injection. In vivo mitochondrial membrane potential in tumor cells was detected by intravenous injection of 50 µL Rhodamine 123 (10 mg/ml, Sigma) and 50 µL of Hoechst 33342. Intravital fluorescence images were acquired with ×25 objective by a two-photon laser scanning microscope (Olympus FVMPE-RS Hybrid).

Mitochondrial superoxide was detected by MitoSOX superoxide indicator (M36008). Cells were incubated with MitoSOX Red (500 nM, M36008) for 15 min in the dark before confocal microscopy (Zeiss LSM 880) and flow cytometry analysis (NovoCyte Quanteon/Advanteon).

### Mitochondrial function assessment

Mitochondrial function was assessed by using Seahorse XF cell mito stress test kit (103015-100, Agilent) according to the manufacturer’s protocol. Briefly, 4000 cells were seeded onto Seahorse XF 96 well cell culture plates for one day, modulators of cellular respiration (oligomycin, FCCP, rotenone and antimycin A) were injected into the wells at indicated time points. Oxygen consumption rate (OCR) was measured by Seahorse XF analyzer (Agilent).

### Subcutaneous and orthotopic tumor model

Immunodeficient BALB/cAnN-nu athymic nude mice (aged 5–8 weeks) were obtained from the Center for Comparative Medicine Research at the LKS Faculty of Medicine, The University of Hong Kong and were housed in individually vented cages (IVCs). IVC cages were maintained under controlled conditions, including consistent temperature, positive air pressure, and filtered air, following a standard 12-hour light/dark cycle. The handling of animals adhered to the guidelines set by the Committee on the Use of Live Animals in Teaching and Research (CULATR) at the University of Hong Kong.

For the subcutaneous xenograft model, mice were injected with 1 × 10^6^ luciferase-expressing U87 cells in the flank region. After 30 days, tumor tissue was collected, weighed, and prepared for further analysis. For the orthotopic xenograft model, a small burr hole was created at 2 mm lateral and 1 mm dorsal to the bregma, followed by the injection of 1 × 10^5^ luciferase-expressing U87 cells into the right striatum using a 26 G Hamilton syringe on a stereotactic apparatus. Tumor growth was measured by bioluminescence measurements using the IVIS Spectrum in vivo imaging system (PerkinElmer) after intraperitoneal injection of the reporter substrate D-luciferin (15 mg/mL). On day 14 post injection, U87-bearing mice were subjected to subcutaneous injection of either vehicle or AMG487 (10 mg/kg) for 10 consecutive days.

### Immunohistochemical staining

For tissue morphology studies, brain specimens were harvested and fixed with 10% formalin solution. Subsequently, 5 μm paraffin tissue sections were prepared for Hematoxylin and Eosin staining. Stained tissue sections were scanned using the Vectra Polaris automated imaging system (PerkinElmer).

### Clinical specimens and survival analysis

Fresh tumor tissues were snap-frozen immediately after surgical resection at the Queen Mary Hospital. Glioma specimens were collected with informed consent from all patients. The study protocol was approved by the Institutional Review Board (Hong Kong West Cluster) of our institute (UW 07-273). Diagnosis and histological classification were confirmed by pathologists according to the World Health Organization (WHO 2021) brain tumor classification system. Glioma patients were stratified into CXCR3^high^ and CXCR3^low^ groups by using the median expression as a cutoff. Survival data were analyzed from TCGA, CGGA, and our clinical database. A Kaplan–Meier survival curve was generated, and a log-rank test was used to assess the statistical difference between CXCR3^high^ and CXCR3^low^ groups.

### RNA sequencing analysis and molecular docking

RNA from xenograft tissue was extracted for PolyA+ mRNAseq. cDNA library was prepared and sequenced on Illumina NovaSeq 6000 at the Center for Genomic Sciences, University of Hong Kong. RNA-seq data were analyzed by the upstream analysis software (FastQC, Fastp, and STAR) to perform quality control, data filtering, and read alignment to obtain the count and FPKM values of each sample. Next, DEGs were detected using the R package ‘DESeq2’, and GO function enrichment was performed using the R package ‘clusterProfiler’. Mitochondrial pathways were further analyzed using the approach previously reported [28]. Transcriptomic data of human glioma samples were downloaded from The Cancer Genome Atlas (TCGA) (https://tcga-data.nci.nih.gov/tcga/) and the CGGA (http://www.cgga.org.cn/) for CXCR3 expression and survival analysis. GBMs were stratified into CXCR3^high^ and CXCR3^low^ groups by using the median CXCR3 mRNA expression as the cutoff. STAT3 mRNA expression was compared between CXCR3^high^ and CXCR3^low^ groups. The CXCR3 protein structure complexed with antagonist AMG487 (8K2W) was collected from the Protein Data Bank (https://www1.rcsb.org/). The 3D view of the molecular docking was visualized using the PyMol software. The intermolecular forces were displayed using MOE software.

### Statistical analysis

The statistical analysis was performed using GraphPad Prism 8.0 Software. Data were presented as mean ± standard deviation or standard error of the mean as indicated. To evaluate the differences between the two groups, a two-tailed unpaired *t* test was employed. For comparisons involving more than two groups, one-way ANOVA was used. A significance level of *p* < 0.05 was considered to be statistically significant.

## Supplementary information

Original Data

Supplementary Table S1

## Data Availability

The datasets generated during and/or analyzed during the current study are available from the corresponding author on reasonable request.
