# Supplementary material for: Non-immune targeting of CXCR3 compromises mitochondrial function and suppresses tumor growth in glioblastoma
Source: Cell Death Discov. 2025 Apr 4;11:143. doi: 10.1038/s41420-025-02449-1 (PMC11971461; doi:10.1038/s41420-025-02449-1)

Raw data for figure 1:

1D)

Labelled clinical specimen 1 in files

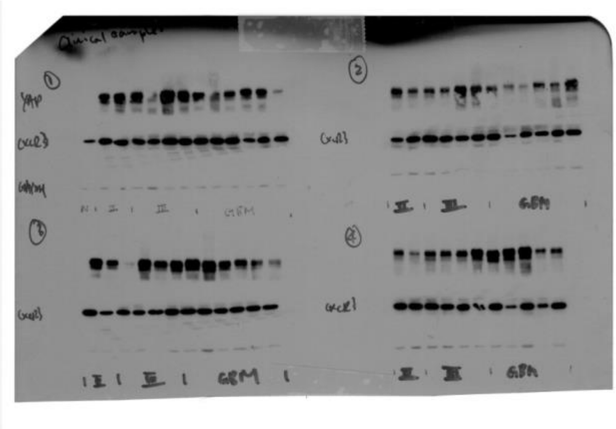

- Labelled clinical specimen 4 in files

Gap for top row

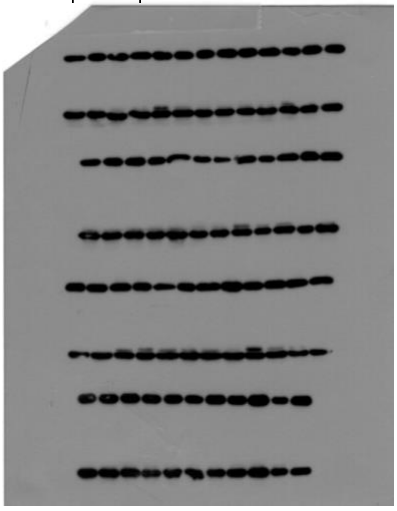

Raw data Figure

4A)

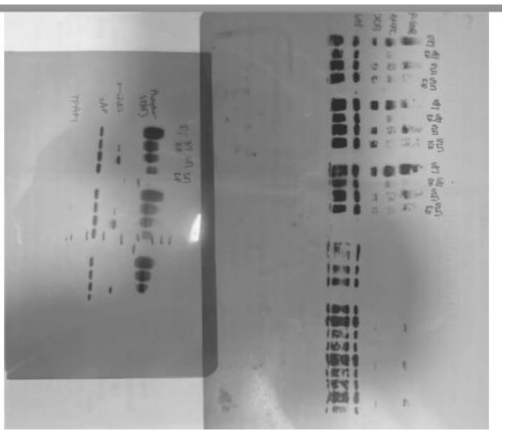

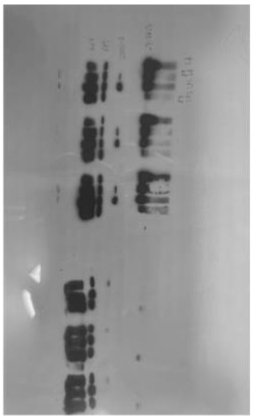

Supplement: Supplementary file 1 — Original Data [file 41420_2025_2449_MOESM1_ESM.pdf]
