# Supplementary material for: Non-immune targeting of CXCR3 compromises mitochondrial function and suppresses tumor growth in glioblastoma
Source: Cell Death Discov. 2025 Apr 4;11:143. doi: 10.1038/s41420-025-02449-1 (PMC11971461; doi:10.1038/s41420-025-02449-1)
Supplement: Supplementary file 2 — Supplementary Table S1 [file 41420_2025_2449_MOESM2_ESM.pdf]

Supplementary Table S1

Table S1. Primer Sequence used in RT-qPCR

Gene of interest	Primer sequence
CXCR3-A	Forward 5’ – CCATGGTCCTTGAGGTGAGTG –3’
	Reverse 5’ – AGCTGAAGTTCTCCAGGAGGG –3’
CXCR3-B	Forward 5’ – TGCCAGGCCTTTACACAGC –3’
	Reverse 5’ – TCGGCGTCATTTAGCACTTG –3’
GAPDH	Forward 5’ – GCTCTCTGCTCCTCCTGTTC –3’
	Reverse 5’ – ACGACCAAATCCGTTGACTC – 3’
